# Low-Temperature Performance and Tribological Properties of Poly(5-*n*-butyl-2-norbornene) Lubricating Oils: Effect of Molecular Weight and Hydrogenation on the Viscosity and Anti-Wear Activity

**DOI:** 10.3390/polym17243333

**Published:** 2025-12-17

**Authors:** Valeriia R. Nazemutdinova, Sergey O. Ilyin, Aleksandr A. Morontsev, Igor S. Makarov, Alyona I. Wozniak, Maxim V. Bermeshev

**Affiliations:** 1 A.V. Topchiev Institute of Petrochemical Synthesis, Russian Academy of Sciences, 29 Leninskiy pr., 119991 Moscow, Russia; nazemutdinova@ips.ac.ru (V.R.N.); s.o.ilyin@gmail.com (S.O.I.); makarov@ips.ac.ru (I.S.M.); wozniak@ips.ac.ru (A.I.W.); 2 Enikolopov Institute of Synthetic Polymeric Materials, Russian Academy of Sciences, Profsoyuznaya str. 70, 117393 Moscow, Russia

**Keywords:** norbornene polymers, metathesis polymerization, synthetic oils, hydrogenated oils, viscosity, friction, wear, tribology

## Abstract

A series of poly(5-*n*-butyl-2-norbornene) oils with controlled molecular weights was synthesized via metathesis polymerization, fully hydrogenated, and characterized in terms of viscosity and tribological performance. In contrast to established lubricant base stocks—such as poly(α-olefins) and multiply alkylated cyclopentanes—these novel norbornene-based polymers remain underexplored, despite their promising anti-wear activity. Based on differential scanning calorimetry (DSC) data, all the synthesized products are amorphous compounds whose thermograms show a single glass transition temperature. The effect of molecular weight and temperature on the viscosity of poly(5-*n*-butyl-2-norbornene) oils was quantified over an extended temperature range, including extra-cold conditions down to −80 °C. The pour points of the oils were determined and can be as low as −66 °C, indicating excellent low-temperature fluidity. The tribological performance of the synthesized oils was evaluated using the four-ball test, with friction coefficient and wear scar diameter measured to assess anti-wear and antifriction properties. The tribological results were benchmarked against commercially available polyalphaolefin (PAO) oils (**PAO-4**, **PAO-20**, and **PAO-80**). Metathesis and hydrogenated poly(5-*n*-butyl-2-norbornene) oils outperform conventional PAOs by up to 67% in wear protection and 30% in friction reduction. These findings establish alicyclic molecular strain as a viable design parameter for next-generation lubricating oils, thereby expanding the toolbox for material development beyond conventional chemical functionalization.

## 1. Introduction

In recent decades, studies of homo- and copolymers containing alkyl-substituted norbornenes as monomer units have attracted increasing attention from researchers [1,2,3]. Although ring-opening metathesis polymerization (ROMP) of norbornene derivatives allows one to obtain products with different architectures and controlled molecular weight [2,4,5,6,7,8], researchers mainly focused on the synthesis of high-molecular-weight products, while liquid polymerization products were considered as side products that were removed by precipitating the target polymer in a poor solvent. However, liquid hydrocarbon oils have long been used as lubricants in addition to mineral and synthetic oils [9].

Unlike synthetic oils, such as esters, phosphate esters, silicones, and fluorocarbons, mineral oils are generally less suitable for use in extreme conditions, for instance, high or low temperatures, high pressure, or aggressive chemical environments [9,10,11], although their lubricating properties can be improved by nanoparticle additives [12]. On the contrary, synthetic oils possess a number of valuable properties, such as low-temperature fluidity, retention of lubricating properties at elevated temperatures, low evaporability, and high chemical and thermal stability [13,14,15]. Typical examples are hydrogenated poly(α-olefins) [16]. It was shown that the operating properties of base oils based on poly(α-olefins) depend on the degree of branching and on the size of the side substituents [14,15,17]. In this respect, poly(alkyl-substituted norbornenes) can be treated as analogues of poly(α-olefins). However, mineral lubricants containing cycloparaffins (naphthenes) are characterized by higher pour points and increased kinetic viscosity at −40 °C than branched poly(α-olefins) [18], as well as by a lower viscosity index (VI) than linear paraffins (the larger the number of fused rings, the lower the VI) [19,20]. However, cycloalkanes with rings of varying size and structural complexity exhibit different rheological properties [21]. Therefore, oils containing cyclic fragments deserve particular and thorough studies aimed at establishing correlations between their structure and properties. Since linear paraffins have the highest pour points, branched paraffins and monocyclic alkanes with alkyl side substituents are most often used as lubricating base oils because of their lower pour points and acceptable VI values [21]. In addition, an increase in the content of naphthenes in the low-viscosity dispersion medium improves the lubricating ability of low-temperature greases [22].

The metathesis-synthesized and then optionally hydrogenated poly(5-*n*-butyl-2-norbornene) oils are also structural analogues of multiply alkylated cyclopentanes (MACs) bearing two or more substituents. When used as lubricants, MACs possess excellent viscosity characteristics, thermal stability, low volatility, and friction-reducing ability [23,24]. These properties enable their widespread use as boundary lubrication agents [25]. The properties of this class of compounds depend on the number and length of hydrocarbon substituents. There is ongoing debate regarding the anti-wear performance of MACs, with studies reporting both pronounced protective effects and limited efficacy under boundary lubrication conditions [23,26,27]. Anyway, these compounds combine the best properties of perfluoropolyethers and synthetic hydrocarbons [23]. Their drawback is a narrow range of operating temperatures and the ease of washing out, which restricts their use as greases in elastohydrodynamic systems [25,28], especially high-vacuum ones [29].

Earlier, we demonstrated that low-molecular-weight metathesis and hydrogenated poly(5-*n*-butyl-2-norbornene) oils used as lubricants have acceptable VI values and strongly improve the wear resistance of steel surfaces [30]. To broaden the potential applications of these oils and to elucidate the influence of molecular weight on their operational performance, a series of polymers spanning a broader molecular weight range than previously studied [30] should be synthesized and characterized. Particular emphasis should be placed on detailed rheological and tribological evaluation. This work aims to study the rheo-tribological behavior of metathesis and hydrogenated poly(5-*n*-butyl-2-norbornene) oils characterized by a degree of polymerization ranging from 5 to 14.

## 2. Materials and Methods

### 2.1. Materials

#### 2.1.1. Initial Reagents

All reactions were carried out under a dry argon atmosphere using standard Schlenk and vacuum-line techniques. THF and 1-hexene were purchased from Komponent-Reaktiv (Moscow, Russia), distilled over sodium under a dry argon atmosphere, and stored under argon. 5-*n*-Butyl-2-norbornene was synthesized by the Diels–Alder reaction at 220 °C as described previously [31]. The fraction of the *exo*-isomer was 25% [32].

Grubbs catalyst of the second generation 3-*bis*-(2,6-trimethylphenyl)-2-imidazolidinylidene]dichloro(phenylmethylene)(tricyclohexylphosphino)ruthenium (Gr2, no less than 98%, Shaanxi Dideu Medichem Co., Baoji City, China), ethyl vinyl ether (no less than 98%, Jiangsu Ambition New Materials Co., Wuxi, China), inhibitor 2,2’-methylene-*bis*(4-methyl-6-*tert*-butylphenol) (Sigma Aldrich, St. Louis, MO, USA), dicyclopentadiene (no less than 96%, Jiangsu Juming Chemical Process Technology Co., Jiangyin, China), hexane (chemically pure grade, Ekos-1, Moscow, Russia) for a chromatographic column, argon (no less than 99.999%, NII KM, Moscow, Russia), hydrogen (no less than 99.999%, Voessen, Moscow, Russia), aluminum oxide 90 neutral (for chromatography, Macherey-Nagel, Düren, Germany), **PAO-4, PAO-20 and PAO-80** polyalphaolefin oils (General Lubricants, Moscow, Russia), balls made from steel “ShKh15” (analog of steel GCr15), and deuterated CDCl_3_ (no less than 99.60%, Cambridge Isotope Laboratories, Tewksbury, MA, USA) were used as received.

#### 2.1.2. ROMP of 5-*n*-Butyl-2-norbornene Initiated by the Second-Generation Grubbs Catalyst

A solution of 28.7 mg (0.034 mmol) of the second-generation Grubbs catalyst in 0.67 mL of THF was added to a mixture of 5-*n*-butyl-2-norbornene (16.52 g, 0.11 mol), 1-hexene (3.70 g, 0.04 mol), and 4.32 mL THF previously prepared in a 100 mL glass reactor. ROMP was carried out for an hour with stirring; the reaction was quenched by adding 1.74 mL of ethyl vinyl ether, filled with the inhibitor. Then the reaction mixture was divided into two parts, and one of them (14 g) was used for hydrogenation. The copolymer from the 12 g reaction mixture was purified by passing through a chromatographic column filled with aluminum oxide, and hexane was the eluent. The resulting solution was refilled with the inhibitor. Low-boiling components were removed under reduced pressure, and the resulting oligomers were dried in vacuum (0.2–0.05 Torr) at 50 °C for 2 h. A transparent colorless oligomer was obtained (8.63 g, yield of 95%). $M_{n}^{\mathrm{GPC}}$ (**M2.5**) = 0.80 kDa, *Ð* = 1.9, *ρ*^20^ (**M2.5**) = 0.888 g/cm^3^. ^1^H NMR spectrum of **M2.5** was identical to the one obtained earlier [30].

#### 2.1.3. Hydrogenation of Metathesis Oligo(5-*n*-butyl-2-norbornene)

A 300 mL autoclave was charged with 16.6 mL (14.0 g) of the reaction mass obtained as a result of metathesis oligomerization of 5-*n*-butyl-2-norbornene, as well as 3.9 mL of triethylamine solution in methanol (the solution was prepared by mixing 0.15 mL of triethylamine with 36 mL of methanol). The autoclave air was replaced with hydrogen by filling the autoclave with gas three times and bleeding off its excess. Before the reaction, the autoclave was filled with hydrogen for the fourth time to 80 bar. Hydrogenation was carried out with constant stirring (500 rpm) under 110 bar and 110 °C for 18 h. The product was purified from residual ruthenium by column chromatography. The product was then recovered by evaporation of the low-boiling fraction under reduced pressure at 40 °C. The obtained hydrogenated oligomer was additionally dried in vacuum (0.2–0.05 Torr) at 70 °C for 2 h. The transparent oligomer was produced (9.4 g, yield of 86%). $M_{n}^{\mathrm{GPC}}$ (**H2.5**) = 0.82 Da, *Ð* = 1.9, *ρ*^20^ (**H2.5**) = 0.877 g/cm^3^. ^1^H NMR spectrum of **H2.5** was identical to the one obtained earlier [30].

### 2.2. Methods

^1^H NMR spectra of the initial and hydrogenated oligo(5-*n*-butyl-2-norbornene) solutions in deuterated CDCl_3_ were recorded with a Bruker Avance DRX 400 spectrometer (Billerica, MA, USA) operating at 400.1 MHz for ^1^H. The proton chemical shifts were determined relative to the residual signal of chloroform (7.26 ppm for ^1^H). Each sample was prepared by dissolving ~40 mg of the polymer in 0.6 mL of the deuterated solvent. To obtain a ^1^H NMR spectrum, four scans of the dissolved sample with a relaxation delay of 10 s were recorded.

Gel permeation chromatography of the initial and hydrogenated oligo(5-*n*-butyl-2-norbornene) was performed with an Agilent 1280 Infinity II installation (Santa Clara, CA, USA) with two series-connected 250 × 4.6 mm PLgel 3 μm MiniMIX-E columns using a refractometric detector. Tetrahydrofuran (no less than 99.9%, Panreac Química SLU, Castellar del Valles, Spain) was used as an eluent; the flow rate was 0.3 mL min^–1^, the sample volume was 15 μL, and the sample concentration was 1 mg ml^–1^. The molecular mass and dispersity were calculated from the calibration dependence of the molecular mass on the retention time, constructed using monodisperse polystyrene standards (Agilent GPC/SEC Calibration Kits, S-L2-10): *M*_p_ = 162 Da, *M*_p_ = 370 Da, *M*_p_ = 580 Da, *M*_p_ = 935 Da, *M*_p_ = 1180 Da, *M*_p_ = 1790 Da, *M*_p_ = 2700 Da, *M*_p_ = 4730 Da, *M*_p_ = 7130 Da, *M*_p_ = 9690 Da. The dependence was linear in the range of 162–9690 Da. The chromatograms were processed using the Agilent GPC/SEC software version A.02.01 build 9.34851 by Agilent Technologies (Santa Clara, CA, USA).

Densities of metathesis and hydrogenated oligomers were determined with a VIP-2MP (Termex, Tomsk, Russia) vibrating-tube densimeter according to the normalized method defined by the norm ASTM D 4052 at 20 °C. The expression *ρ* = *A* + *Bτ*^2^ was used to obtain the densities of the them, where *τ* is the oscillation period, while *A* and *B* are coefficients determined from calibration at 298.15 K by known densities and oscillation periods of ambient air, ultrapure water, and liquid density standard—1000 (0.998 g/cm^3^, produced and certified by ECROSKHIM Co., St. Petersburg, Russia). The temperature was maintained by the built-in thermostat; the uncertainty in the temperature was 0.02 °C. The standard deviation for the measurement of the solution density was 0.001 g/cm^3^.

Differential scanning calorimetry (DSC) was performed with a TA 4000 device (Mettler Toledo, Greifensee, Switzerland) at a temperature variation rate of 20 °C/min in the range from −140 to 100 °C under argon (99.99%, Akron, Moscow, Russia; gas flow rate was 70 mL/min). The glass transition temperature (*T*_g_) was determined from the data obtained after the repeated heating of the sample. The measurement results were processed using the STARe software 16.10 by Jeol (Tokyo, Japan) supplied with the device. The measurement accuracy was as follows: *ΔT* = ±0.3 °C and *ΔH* = ±1 J/g.

Viscosity properties were studied using a stress-controlled rotational rheometer (Discovery HR-30, TA Instruments, New Castle, DE, USA) equipped with a cone–plate unit featuring a 40 mm cone diameter and a 2° cone/plate angle. The temperature dependences of viscosity were determined at a temperature decrease rate of 3 °C/min using a constant shear stress of 1, 3, 10, or 100 Pa, depending on the viscosity of the sample under study, while merging the resulting curves. The relative error in measuring the rheological values did not exceed 5%.

Tribological tests were conducted on the same rheometer using a four-ball method. The balls were made of high-carbon chromium-bearing steel ShKh15 (Young’s modulus of 211 GPa) with a diameter of 6.5 mm. Before tests, the balls were degreased with acetone, and then three balls were placed stationary in a cylindrical cell and flooded with the oil under study. The upper ball pressed the lower three balls with a force of 50 N, corresponding to a Hertzian maximum contact pressure of approximately 1200 MPa. The angular velocity of the upper ball was 100 rad/s, corresponding to a linear velocity at the wear points of 0.188 m/s. The test duration was 60 min at 75 °C, and each test was repeated under identical conditions to assess reproducibility. The coefficient of friction was calculated as follows [30]:

(1) $$f=\;\frac{\sqrt{2}M}{rF},$$

where *M* is the torque, *r* is the ball radius, and *F* is the applied force. Averaging of *f* was conducted using data from the last 20 min of the tests. After that, without shifting the balls relative to each other to keep their lapping, the Stribeck curves [33] were measured with a stepwise increase in sliding speed from 1.5 × 10^−5^ to 0.56 m/s at 50 N and 75 °C. Then, the wear scar diameter of the balls was measured on an optical microscope by averaging the values for the three lower balls.

Scanning electron microscopy (SEM) of the ball surface morphology was studied using a JSM-6000PLUS scanning electron microscope (JEOL, Tokyo, Japan) equipped with an energy dispersive X-ray spectrometer (EDX, JED-2300, JEOL, Tokyo, Japan). A thin layer of conductive material (gold) was preliminarily sprayed onto the surface of the sample. Then, samples were attached to the surface of an aluminum table using double-sided carbon tape. The study was carried out in the secondary electron mode at an accelerating voltage of 5^−10^ keV in a vacuum of 10^−2^ Pa.

## 3. Results and Discussion

### 3.1. Metathesis Polymerization of 5-n-Butyl-2-norbornene and Subsequent One-Pot Hydrogenation of the Resulting Polyenes

**Scheme 1:**
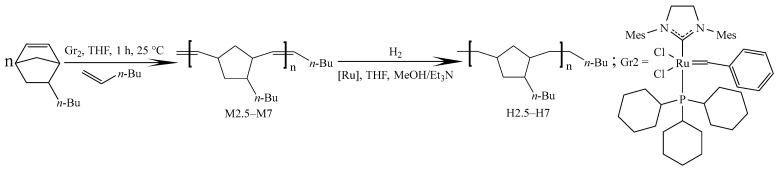
Synthesis of metathesis poly(5-*n*-butyl-2-norbornenes) and their subsequent hydrogenation.

The starting metathesis (**M2.5**–**M7**) and hydrogenated (**H2.5**–**H7**) poly(5-*n*-butyl-2-norbornene) oils (Scheme 1) were obtained by analogy with the previously developed procedure [30] at different ratios of the starting reagents (Table 1). The molecular weight was controlled by adding hexene-1 as a chain transfer agent (CTA) to the reaction mixture.

Both the metathesis and hydrogenated oils were purified by column chromatography. The final metathesis and hydrogenated poly(5-*n*-butyl-2-norbornenes) were obtained in yields from 80 to 98%. The structures of the products were confirmed by ^1^H NMR spectroscopy. All the oils obtained were also characterized by GPC. The average degree of polymerization (*P*) varied from 3 to 14 monomer units, and the dispersity varied in the range of 1.7–2.0. An increase in the amount of hexene-1 in the reaction mixture caused a decrease in the molecular weight of the metathesis products. The synthesis of the initial metathesis products in the presence of a small amount of CTA at room temperature was accompanied by an intense exothermic effect. To reduce the release of excess heat, a smaller amount of catalyst was added, and the reaction mass was also cooled with ice in a water bath for the first 30 min of the reaction. This stabilized the polymerization process on the one hand and led to some increase in the molecular weight on the other. The latter can be due to the higher reactivity of the strained double bond in the norbornene derivative compared to that of the terminal double bond in hexene-1 and, as a consequence, to some predominance of the ring-opening reaction over the chain transfer reaction. Subsequent one-pot hydrogenation afforded products where the backbone carbon–carbon double bonds were fully converted to single bonds. Hydrogenation had almost no effect on the changes in the molecular weight (see Table 1, **M2**–**M7** vs. **H2**–**H7**).

The metathesis and hydrogenated samples had similar density (Table 2), which somewhat increased with increasing degree of polymerization. Metathesis oils were characterized by higher fluidity than hydrogenated oils with the same molecular weight.

### 3.2. Thermophysical Properties of Poly(5-n-butyl-2-norbornene) Oils

According to TGA data, the 5% weight loss temperatures of the synthesized metathesis and hydrogenated poly(5-*n*-butyl-2-norbornene) oils exceed 300 °C. This is indicative of the high thermal stability of the synthesized polymeric products.

**Figure 1:**
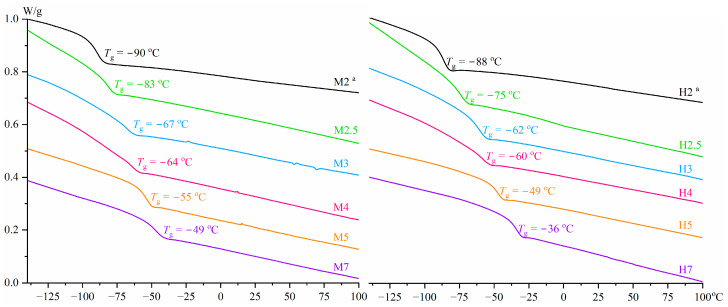
DSC curves of metathesis (**M2**–**M7**) and hydrogenated (**H2**–**H7**) poly(5-*n*-butyl-2-norbornene) oils with different molecular weight. ^a^ Data were obtained previously and adapted with permission from [30], Energy and Fuels, 2025. The designations correspond to the products listed in Table 1. All oils are amorphous; no melting or cold-crystallization events are detected—only a glass transition is observed.

Based on DSC data (Figure 1), all the synthesized samples were amorphous compounds whose thermograms show a single glass transition temperature (*T*_g_). This agrees with the previously published results obtained for the same-type products having lower molecular weights [30]. The *T*_g_ values increase in parallel with the degree of polymerization.

The glass transition temperatures of low-molecular-weight polymers (*T*_g_) and those of high-molecular-weight ones (*T*_g,∞_) are related by the Flory–Fox equation [34,35] as follows:

(2) Tg=Tg,∞−kMn,

where *k* is an empirical constant, which depends on the density, free volume, and thermal expansion of the low- and high-molecular-weight polymers.

**Figure 2:**
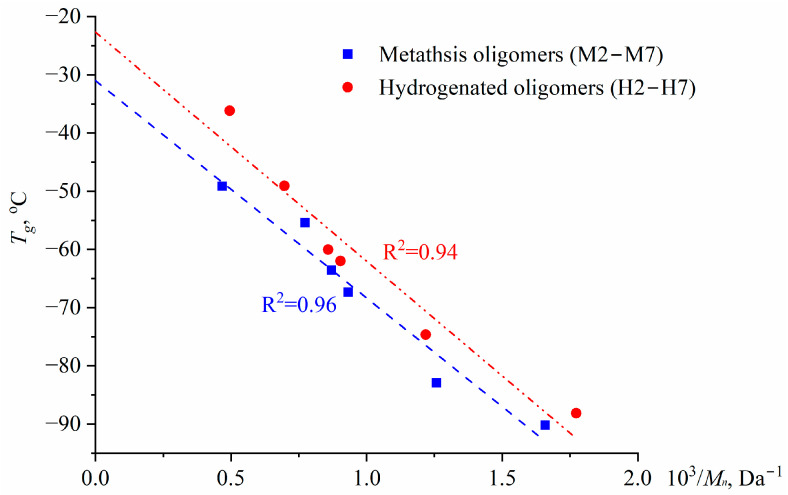
Glass transition temperature of metathesis (**M2**–**M7**) and hydrogenated (**H2**–**H7**) norbornene oils plotted vs. their molecular weight in coordinates of the Flory–Fox equation. Data for **M2** and **H2** were obtained previously [30]. Adapted with permission from [30], Energy and Fuels, 2025.

The plots of *T*_g_ vs. molecular weight (*M*_n_) of the metathesis (**M2**–**M7**) and hydrogenated (**H2**–**H7**) oils (Figure 2) are straight lines with the coefficient of determination *R*^2^ = 0.94–0.96. These coefficients are lower than those we have obtained recently for poly(5-*n*-butyl-2-norbornene) oils after their fractionation [30]. The reason is that the studied oils are characterized by a wider range of dispersity values (*Ð* = 1.6–2.0) compared to the dispersity of the products isolated by preparative chromatography [30]. Extrapolation of *T*_g_ values to 1/*M*_n_ = 0 enables estimation of the limiting glass transition temperatures for high-molecular-weight analogues of the same chemical structures: *T*_g_ = −31 °C for the high-molecular poly(5-*n*-butyl-2-norbornene) and *T*_g_ = −23 °C for its hydrogenated counterpart, which is very close to the values determined previously [30].

### 3.3. Rheological Properties of Poly(5-n-butyl-2-norbornene) Oils

**Figure 3:**
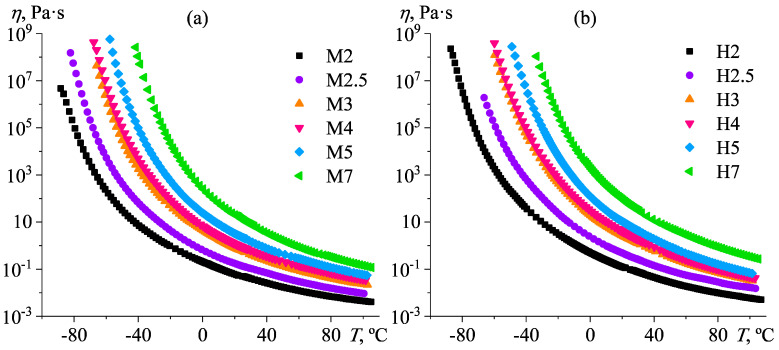
Temperature dependences of viscosity for poly(5-*n*-butyl-2-norbornene) oils before (**a**) and after (**b**) hydrogenation. Data for **M2** and **H2** were obtained previously [30]. Adapted with permission from [30], Energy and Fuels, 2025.

**Figure 4:**
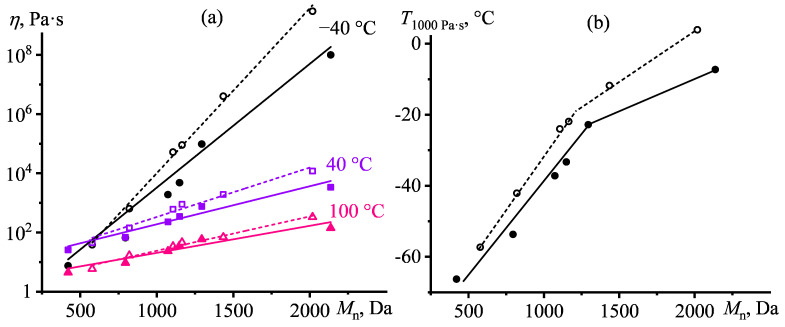
Viscosity (**a**) and pour point (**b**) as a function of molecular weight for poly(5-*n*-butyl-2-norbornene) oils before (filled symbols and solid lines) and after (open symbols and dashed lines) hydrogenation.

The viscosities of the synthesized poly(5-*n*-butyl-2-norbornene) oils increase gradually with decreasing temperature, both for the metathesis oils (Figure 3a) and their hydrogenated counterparts (Figure 3b). The smooth rise in viscosity to about 10^7^–10^9^ Pa·s indicates a gradual glass transition [36] and the absence of crystallization, which would otherwise cause a sharp increase in viscosity accompanied by the emergence of a yield stress [37]. An increase in the molecular weight linearly elevates the oil viscosity at low, moderate, and high temperatures of −40, 40, and 100 °C in semi-logarithmic coordinates (Figure 4a). Notably, regardless of temperature, the viscosities of the hydrogenated oils are higher than those of the initial metathesis poly(5-*n*-butyl-2-norbornene) oils at comparable molecular weights. Moreover, the lower the temperature, the larger the increase in viscosity with increasing molecular weight. No significant correlation exists between viscosity and dispersity (*Ð*) of the oil molecules (*p*-value > 0.1; here, *p* < 0.01 indicates good evidence that the correlation does exist, *p* < 0.05 is for moderate evidence, *p* < 0.1 is for weak evidence, while *p* > 0.1 points to the fact that there is no evidence) [38].

The viscosity of poly(5-*n*-butyl-2-norbornene) oils increases exponentially with molecular weight, as evidenced by the linear dependence of log*η* on *M*_n_ (Figure 4a). This behavior is characteristic of unentangled oligomers with restricted internal rotation, where viscosity is governed by the cumulative energy barrier to segmental motion rather than by chain reptation. Consequently, the Rouse/Zimm power law (*η* ~ *M*_n_*^α^*) is not applicable. Instead, the dependence follows *η* ~ exp(*βM*_n_), where *β* reflects the torsional rigidity of the backbone. The steeper slope for hydrogenated oils (*β* = 0.0039 Da^−1^ vs. 0.0029 Da^−1^ for metathesis oils at 40 °C) quantitatively confirms the stiffening effect of double-bond saturation, which is consistent with the concurrent increase in *T*_g_ (Figure 2).

A decrease in temperature and the accompanying rise in viscosity cause the nominal loss of fluidity of glass-forming oils, defined as the point at which the viscosity reaches approximately 1000 Pa·s [17,39]. In our case, the hydrogenated oils exhibit a higher pour point at the same molecular weight (Figure 4b), which is due to their higher viscosity. The pour point increases linearly with the molecular weight of the oil up to 1100–1300 Da (a polymerization degree of 7–8). Beyond this range, the rate of pour point elevation decreases. Furthermore, the pour point exhibits a strong negative correlation with dispersity, as the Pearson linear correlation coefficient (*ρ*_P_) equals −0.73 at *p* < 0.01 (where *ρ*_P_ = 0 indicates no correlation, while 1 and −1 represent an ideal positive and negative correlation, respectively [40,41]. In other words, a broader molecular weight distribution is preferable for improved low-temperature fluidity.

**Figure 5:**
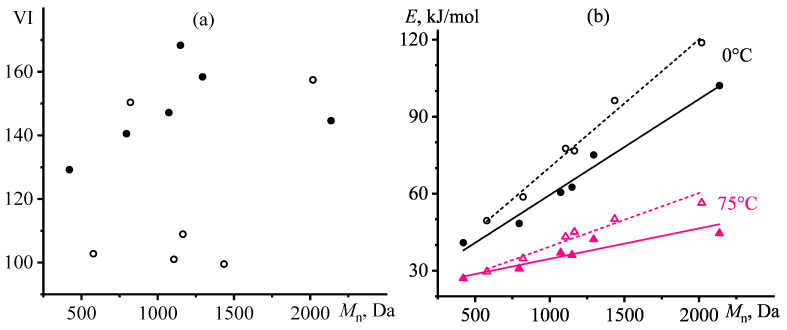
Viscosity index (**a**) and flow activation energy (**b**) as a function of molecular weight for poly(5-*n*-butyl-2-norbornene) oils before (filled symbols and solid lines) and after (open symbols and dashed lines) hydrogenation.

The sensitivity of oil viscosity to temperature variations is often described using the VI values that range from 0 to 100 for standard naphthenic and paraffinic oils, respectively, whose viscosities exhibit stronger or weaker temperature dependence [42]. In our case, the VI of poly(5-*n*-butyl-2-norbornene) oils does not correlate with their molecular weight (Figure 5a). An alternative and more scientifically substantiated measure of the temperature dependence of viscosity is the flow activation energy, as defined by the Frenkel–Andrade equation [43] as follows:

(3) $$\eta =\;Ae^{\frac{E}{RT}},$$

where *A* is the fitting parameter, *T* is the absolute temperature, *E* is the flow activation energy, and *R* is the gas constant. This approach provides a physically meaningful quantification of the energy barrier to molecular motion in the liquid state.

Since the test temperatures are in relative proximity to the glass transition temperatures of poly(5-*n*-butyl-2-norbornene) oils, their flow activation energy is temperature dependent, while the viscosity increases sharply upon cooling [44]. Nevertheless, calculations of the activation energy at low and high temperatures (0 and 75 °C, respectively) reveal that it increases linearly with increasing molecular weight (Figure 5b). Thus, the activation energy of flow—being a physically grounded measure of the temperature dependence of viscosity—is a more reliable parameter than the viscosity index, as was noted in the previous studies [30,42]. In the case of hydrogenated oils, the flow activation energy increases more steeply with the molecular weight, consistent with the trends observed for viscosity and pour point (Figure 4). Moreover, the lowering of temperature intensifies the rate of increase in the activation energy, mirroring the enhanced sensitivity of viscosity to molecular weight at lower temperatures (Figure 4a). In addition, the activation energies at both 0 °C and 75 °C demonstrate a strong negative correlation with molecular dispersity (*ρ*_P_ = −0.66 or −0.73 at *p* < 0.05). This fact indicates that a lower variation in viscosity with temperature (i.e., lower *E*) is associated with a broader molecular weight distribution (higher *Ð*). In practical terms, improved temperature–viscosity stability can be achieved by tailoring the dispersity of the poly(5-*n*-butyl-2-norbornene) chains.

The viscosity of oils at low and high temperatures determines their intended application, which can be evaluated according to standards established by the Society of Automotive Engineers (SAE), such as SAE J300 for engine oils and SAE J306 for axle and gear oils. In our case, poly(5-*n*-butyl-2-norbornene) oils with molecular weights below 1100 Da are suitable as winter-grade engine oils, with the minimum operating temperatures ranging from −10 °C (25W, Table 2) to −35 °C (0W), and capable of withstanding summer temperatures up to 50 °C (e.g., 25W-50) [45]. The viscosity of higher-molecular-weight poly(5-*n*-butyl-2-norbornene) oils is too high for use as engine oils. However, all the studied oils meet the criteria for gear oils according to SAE J306, with winter serviceability down to −55 °C (70W) and the ability to perform under high-torque conditions and elevated temperatures (e.g., 85W-250 and 250). Table 3 summarizes the rheological characteristics of the poly(5-*n*-butyl-2-norbornene) oils.

### 3.4. Tribological Properties of Poly(5-n-butyl-2-norbornene) Oils

**Figure 6:**
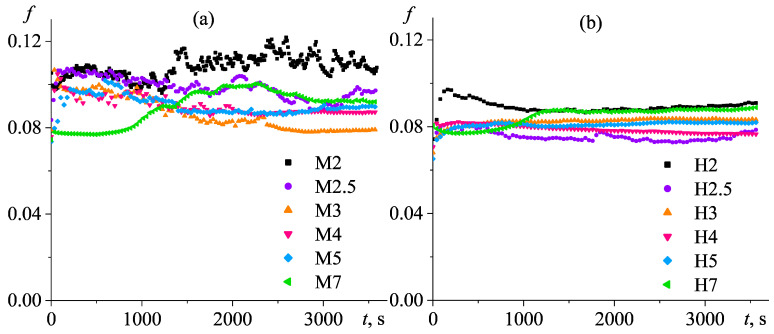
Friction coefficient as a function of time during four-ball wear tests using poly(5-*n*-butyl-2-norbornene) oils before (**a**) and after (**b**) hydrogenation.

In addition to specific viscosity characteristics, effective lubricating oils must minimize friction and wear between contacting surfaces. Four-ball friction tests on poly(5-*n*-butyl-2-norbornene) oils reveal that hydrogenated oils provide more stable friction performance, because the friction coefficient exhibits smaller fluctuations with time when these oils are used (Figure 6b vs. Figure 6a). In the case of non-hydrogenated oils, the observed friction variations may arise from their reactivity at residual double bonds—likely more pronounced in the lowest-molecular-weight oil (**M2**), which also exhibits higher friction (Figure 6a). Hydrogenation consistently reduces the friction coefficient (*f*, Table 3) and the wear scar diameter (*D*_scar_). The lowest friction and wear are observed for oils with intermediate molecular weights of approximately 1100–1400 Da. However, no significant correlations are found between the molecular weight or dispersity and either friction or wear (*p* > 0.1). The friction coefficient and the wear scar diameter demonstrate a weak linear correlation (*ρ*_P_ = 0.56 at *p* < 0.1), which is expected because reduced friction generally leads to less wear. Surprisingly, there are no significant correlations between friction, wear, and key oil indexes such as the molecular characteristics, viscosity, or flow activation energy. However, wear shows a strong linear correlation with the VI values (*ρ*_P_ = 0.77 at *p* < 0.01), which itself does not correlate with any other parameter, even with the flow activation energy (*p* > 0.1). The lack of correlation between viscosity and friction suggests a boundary or mixed lubrication regime, where steel surfaces intermittently come into direct contact, leading to wear. The lubrication regime is governed by the load applied and the sliding speed between surfaces, allowing the transition between lubrication regimes to be analyzed via Stribeck curves—plots of the friction coefficient as a function of sliding speed [33].

**Figure 7:**
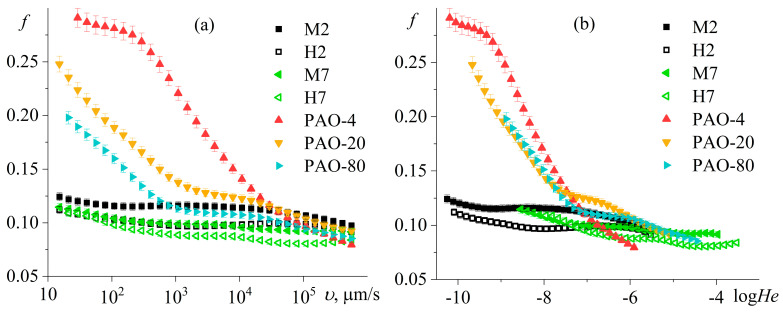
Stribeck curves as a function of sliding speed (**a**) or Hersey number (**b**) for poly(5-*n*-butyl-2-norbornene) and PAO oils.

The Stribeck curves of all poly(5-*n*-butyl-2-norbornene) oils are similar (Figure 7a), characterized by the absence of distinct mixed and boundary friction regimes, i.e., the regions where the friction coefficient typically increases noticeably and then levels off as the sliding speed decreases. In contrast, these regions are clearly observed when polyalphaolefin (PAO) oils are tested as reference samples (Table 4). For the higher-viscosity **PAO-20** and **PAO-80**, well-defined mixed lubrication regimes are evident at sliding speeds below 1000–2000 μm s^–1^. In the case of the low-viscosity **PAO-4**, the Stribeck curve shows transitions from the mixed to the boundary lubrication regime at sliding speeds below 200 μm s^–1^. No such transitions are observed for the polybutylnorbornene oils, whose friction coefficient increases only slightly as the sliding speed decreases. This behavior may indicate a stable hydrodynamic or elastohydrodynamic lubrication regime across the entire speed range, with minimal surface contact and excellent lubricating film formation, even at very low sliding speeds.

At the same time, all tested oils have different viscosities that influence the lubrication regime. The effect of viscosity on the friction coefficient can be eliminated using the dimensionless parameter termed the Hersey number [46] as follows:

(4) $$He=\;\frac{\eta \upsilon L}{F},$$

where *F* is the force applied to the upper ball (50 N), and *L* is the bearing length of the circle circumscribed on the upper ball by the three lower balls with the same ball radii *r* ($L=\;\frac{4\pi r}{\sqrt{3}}$). Replotting the Stribeck curves as a function of the Hersey number does not fundamentally change the picture: poly(5-*n*-butyl-2-norbornene) oils still lack a distinct boundary and mixed friction regions, in contrast to the PAO oils (Figure 7b). However, there is also no clear hydrodynamic lubrication region, where the friction coefficient would gradually increase with increasing Hersey number. Thus, poly(5-*n*-butyl-2-norbornene) oils provide lubrication for sliding steel surfaces in some intermediate regime characterized by weak dependence on the Hersey number (i.e., on the applied load and sliding speed), suggesting consistent film performance across a wide range of operating conditions.

Before obtaining the Stribeck curves, the steel balls were run-in against each other at a sliding speed of 1.9 × 10^5^ μm/s for one hour (as shown in Figure 6). During this period of intense friction, a tribofilm formed on the wear surfaces, differing in nature for the PAO and poly(5-*n*-butyl-2-norbornene) oils. For the PAO oils, this tribofilm appears to be unstable and prone to compositional changes under different friction conditions. In contrast, the tribofilm formed by poly(5-*n*-butyl-2-norbornene) oils is either highly durable (persisting once formed) or readily regenerated under lower sliding speeds and associated local heating. Determining which of these two hypotheses is more accurate is unfeasible because running on pristine surfaces at low sliding speeds would require an impractical amount of time for their lapping and proper experimental conditions. However, the presence of a wear scar indicates surface abrasion and, consequently, continuous renewal of the tribofilm during testing, thus suggesting a combination of rapid tribofilm formation and enhanced stability. Regardless of the exact nature of the poly(5-*n*-butyl-2-norbornene)-derived tribofilm, it provides steel surfaces with a lower friction coefficient at high sliding speeds compared to the PAO oils (0.075–0.111 vs. 0.086–0.104, Table 3 and Table 4, respectively), and the advantage increases twofold and more at lower sliding speeds (see Figure 7). In particular, the wear scar diameter is significantly smaller when using poly(5-*n*-butyl-2-norbornene) oils (*D*_scar_ = 118–359 μm vs. 352–407 μm), which confirms the superior wear resistance of the formed tribofilm. Both the ease of tribofilm formation and the very fact of its appearance may be due to the chemical structure of the poly(5-*n*-butyl-2-norbornene) oils—specifically, the potentially higher reactivity of the norbornene rings compared to that of the branched poly(α-olefin) chains under conditions of high local pressures and temperatures. In such environments, the generated radicals may react with metal atoms in the steel surface, which is also prone to rapid oxidation due to elevated temperatures and ambient oxygen. In other words, the poly(5-*n*-butyl-2-norbornene) oils may participate in surface-active processes that enhance lubrication performance through in situ tribochemical reactions.

### 3.5. Composition and Morphology of Wear Surfaces

**Figure 8:**
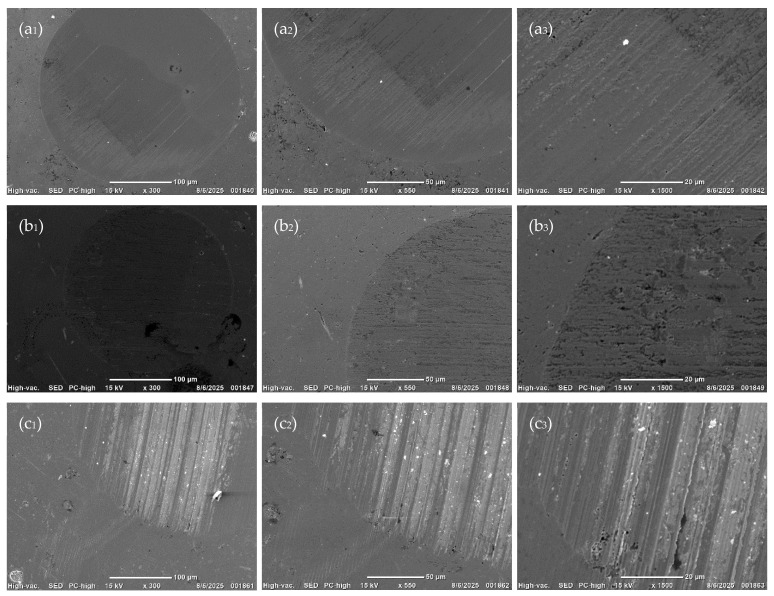
Wear scar morphologies at different magnifications after four-ball tests in the presence of **M7** (**a_1_**–**a_3_**), **H7** (**b_1_**–**b_3_**), and **PAO-4** (**c_1_**–**c_3_**).

Prior to studying the surface morphology of the balls exposed to four-ball friction tests, the balls were washed with hexane and dried. Based on the SEM images, the surface morphology of the tested balls was characterized by a wear scar, which was dependent on the nature of the lubricant (Figure 8). For instance, in the presence of **M7** and **H7**, the wear surface was smooth and had clear edges without signs of abrasive wear and plastic deformation (Figure 8(a_1_–a_3_,b_1_–b_3_)). There are shallow and rare scratches along the sliding direction. Probably, they are situated in the interior of the protective tribofilm, which is formed at the ball contact area and reduces friction and wear. Replacement of poly(5-*n*-butyl-2-norbornene) oil by polyalphaolefin oil (e.g., **PAO-4**) led to significant changes in the character of surface wear. Numerous closely spaced scratches and rough edges are indicative of abrasive wear and plastic deformation during friction in the presence of **PAO-4**, which does not prevent the formation of a heavily damaged worn surface. Qualitative differences in the wear surface morphology confirm the excellent anti-wear properties of the synthesized metathesis and hydrogenated poly(5-*n*-butyl-2-norbornene) oils compared to **PAO-4**. Based on the energy-dispersive X-ray spectroscopy (EDS) data, the atomic and weight compositions of the surfaces within and outside the wear zone differ significantly (Table 5, Figures S1–S4). In particular, the proportion of carbon increased, and oxygen was detected on the surface of the balls after the tests. We assume that the oxygen appeared as a result of metal oxidation by atmospheric oxygen under the action of heat produced by the friction forces. The abrasive surface tested in the presence of **PAO-4** contained the lowest amount of oxygen, which can be due to the higher intensity of surface abrasion in this case and, as a consequence, to renewal of the surface followed by a decrease in the content of oxygen. Improved anti-wear properties of **M7** and **H7** prevented the removal of the oxidized upper layer of the metal, which led to an increase in the proportion of oxygen. In addition, the proportion of carbon increased significantly during the abrasion process. The highest proportion of carbon was detected on the surface tested in the presence of **PAO-4**. Probably, intense metal abrasion led to the appearance of numerous scratches, valleys, and roughness, as compared to the untreated surface or to the surface worn in the presence of the **M7** and **H7** oils. High surface wear facilitated penetration of the hydrocarbon lubricant into surface irregularities and prevented washing out of the lubricant from grooves and valleys, which manifested itself as an increase in the proportion of carbon.

The combined analysis of friction coefficients (Table 3), SEM micrographs (Figure 8), and EDS data (Table 5) reveals a clear mechanistic distinction between poly(5-*n*-butyl-2-norbornene) and PAO lubricants. Poly(5-*n*-butyl-2-norbornene) oils exhibit lower friction coefficients (as low as *f* = 0.075 for **H2.5**) and generate smooth wear scars with shallow, parallel scratches (Figure 8(a_1_–a_3_,b_1_–b_3_)), consistent with mild adhesive wear under effective mixed lubrication, where boundary interactions at asperity contacts dominate wear behavior. The elevated oxygen (4.4–5.1 wt%) and moderate carbon (2.1–3.1 wt%) content on these surfaces (Table 5) suggest the in situ formation of a durable, organometallic-rich tribofilm, likely involving oxidation of freshly exposed steel and adsorption or tribochemical reaction of the strained cyclopentane rings. In contrast, **PAO-4** produces the largest wear scar among all tested lubricants (407 ± 14 µm) and exhibits severe abrasive wear morphology with deep, densely spaced scratches and plastic deformation (Figure 8(c_1_–c_3_)), despite a moderate mean friction coefficient (*f* = 0.104, Table 4). This dissociation between friction level and wear resistance indicates that **PAO-4** fails to maintain a protective boundary film under the applied test conditions, leading to direct asperity contact and material removal. Its low oxygen content (0.1 wt%) reflects rapid surface renewal and suppression of oxide layer accumulation, whereas the high carbon content (4.5 wt%) points to mechanical entrapment of hydrocarbon fragments in wear valleys rather than the formation of a chemisorbed tribofilm. Collectively, these findings support the hypothesis that the superior anti-wear performance of poly(5-*n*-butyl-2-norbornene) oils stems not merely from their viscosity or ability to reduce friction, but from chemically active boundary lubrication enabled by their strained cyclic architecture.

### 3.6. Origin of the Superior Anti-Wear Performance of Poly(5-n-butyl-2-norbornene) Oils

The tribological superiority of poly(5-*n*-butyl-2-norbornene) oils over conventional PAOs cannot be rationalized solely by rheological parameters (e.g., viscosity or VI), as evidenced by the lack of correlation between wear and bulk properties (Section 3.4). Instead, a combined analysis of friction dynamics (Figure 6 and Figure 7), wear morphology (Figure 8), and surface composition (Table 5) points to a chemically enabled boundary lubrication mechanism, driven by the unique molecular architecture of the repeating unit (Scheme 1). Each monomer contains a deformed cyclopentane ring constrained by a bridge: a vinylene (–CH=CH–) unit in metathesis oils (**M2**–**M7**) and an ethylene (–CH_2_–CH_2_–) unit in hydrogenated oils (**H2**–**H7**). Although fully saturated, the ethylene-bridged cyclopentane structure retains significant local steric strain (~25–30 kJ/mol [47]), forcing bond angles to 95–100° and enhancing *σ*-bond polarizability, particularly at the bridgehead carbons. In this system, the 5-*n*-butyl substituent can contribute to low glass transition temperatures and surface wetting, but does not participate directly in interfacial bonding. Under tribocontact conditions with high local temperatures (can be more than 1000 °C [48,49,50]) and extreme pressure (≈1.2 GPa), enhanced polarizability facilitates pressure-assisted charge transfer to Fe 3d orbitals, thereby promoting the formation of a thin Fe–O–C-rich protective surface layer. In contrast, PAO chains lack both strain and directional *σ*-orbitals, limiting them to physisorption.

Quantitative analysis of the EDS data (Table 5) supports this interpretation. First, the O/Fe atomic ratios for **H7** and **M7** (0.20 and 0.16, respectively) are 50 times higher than for **PAO-4** (0.0037), despite all measurements being performed under identical conditions. Second, the C/O ratios for **H7** and **M7** are low (≈0.66–0.79), indicating co-localized carbon and oxygen consistent with chemisorbed species, unlike **PAO-4** (C/O ≈ 60), where carbon is mechanically trapped in deep valleys. While the obtained O/Fe ratios are lower than the theoretical O/Fe = 1.33 for bulk Fe_3_O_4_, this is expected for nanoscale surface films: the EDS signal originates from a depth of several hundred nanometers, thus integrating contributions from both the tribofilm and the underlying steel. Simple mixing-model estimates suggest an effective tribofilm thickness of 80–150 nm. In contrast, the near-undetectable oxygen for **PAO-4** confirms continuous oxide removal under abrasive wear.

This mechanism explains the unique Stribeck behavior (Figure 7): the friction coefficient remains low and stable even at Hersey numbers < 10^−9^, where conventional oils enter the boundary regime. Our interpretation is that tribofilm regeneration locally outruns asperity damage, i.e., the interfacial reaction between poly(5-*n*-butyl-2-norbornene) and steel is kinetically favored over wear processes. This hypothesis is corroborated by the mild adhesive-wear signatures observed under mixed-lubrication conditions (Figure 8(a_1_–a_3_,b_1_–b_3_)): shallow, subsurface scratches indicate that material removal is mitigated by a persistent tribofilm, consistent with effective boundary protection. In contrast, **PAO-4**, lacking reactive sites, cannot replenish the surface film, leading to runaway abrasive wear once the initial oxide is removed. Furthermore, the smooth wear scars (Figure 8(a_1_–a_3_,b_1_–b_3_)) and stable coefficient of friction (Figure 6b), particularly evident for hydrogenated oils, indicate that ethylene-bridged units achieve an optimal balance: sufficient *σ*-bond lability for in situ organometallic tribofilm formation, yet high enough chemical stability to resist degradation. This contrasts with both the more reactive but less durable vinylene-bridged analogues (M-series) and the inert linear chains of PAOs.

Thus, the superior anti-wear efficacy of poly(5-*n*-butyl-2-norbornene) oils is not paradoxical but mechanistically coherent: it stems from a precise balance between chemical lability, which enables tribofilm formation, and thermal stability, which prevents bulk degradation. This combination is rarely achieved in conventional hydrocarbon lubricants. Crucially, the required reactivity emerges not from traditional functional groups, but from the mechanically amplified polarizability inherent to geometrically constrained, strained alicyclic motifs. This insight establishes a new design principle for next-generation boundary lubricants operating under mixed and boundary lubrication conditions, where molecular topology, rather than conventional chemistry alone, governs interfacial activity and performance.

## 4. Conclusions

The metathesis and hydrogenated poly(5-*n*-butyl-2-norbornene) oils with different molecular weights in the range from 0.56 to 2.14 kDa (*Ð* = 1.6–2.0) were obtained for the first time. Based on the DSC data, all the synthesized products are amorphous compounds whose glass transition temperatures increase in parallel with molecular weight in accordance with the Flory–Fox equation. The logarithmic viscosity of both metathesis and hydrogenated samples increases linearly with increasing molecular weight. Hydrogenated samples are characterized by higher viscosities and pour points compared to the initial metathesis poly(5-*n*-butyl-2-norbornene) oils. The flow activation energy increases linearly with the molecular weight of the synthesized oils. The high-fluidity products have nearly equal densities. Depending on molecular weight and whether hydrogenation is performed, the poly(5-*n*-butyl-2-norbornene) oils exhibit pour points ranging from 4 °C to −66 °C, enabling selection of a specific oil with the required viscosity for various temperature conditions—even extremely low ones.

From the results of four-ball friction tests, it follows that the metathesis and hydrogenated poly(5-*n*-butyl-2-norbornene) oils significantly reduce friction and wear compared to commercially available polyalphaolefin oils (**PAO-4**, **PAO-20**, and **PAO-80**). Hydrogenated poly(5-*n*-butyl-2-norbornene) oils provide more stable friction characteristics owing to a smaller range of variation in the friction coefficient. The molecular weight and dispersity have little effect on ball friction and wear. According to the EDS data, the wear surface is oxidized by atmospheric oxygen. The proportion of oxygen on the worn steel surface in the presence of the **M7** and **H7** oils is 40 times higher than in the presence of **PAO-4**. This is probably due to more intense abrasion and renewal of the ball surface in the latter case. The balls tested in the presence of the **M7** and **H7** oils have smooth wear surfaces, indicating adhesive wear. Replacement of these oils by **PAO-4** leads to severe damage to the friction surfaces, which can be due to abrasive wear and plastic deformation. Poly(5-*n*-butyl-2-norbornene) oils reduce wear scar diameter by 50–67% and friction coefficient by 10–30% relative to polyalphaolefin reference oils under identical test conditions. In this case, the superior mixed-to-boundary lubrication performance stems not from functional groups or unsaturation, but from the mechanically amplified polarizability of *σ*-bonds in geometrically constrained cyclopentane units—a design principle wherein molecular topology, rather than chemistry alone, governs interfacial reactivity. This work thus establishes a new paradigm for synthetic lubricants: molecular strain-activated boundary lubrication from fully saturated hydrocarbons.

## Figures and Tables

**Table 1:** Molecular weight characteristics of metathesis and hydrogenated poly(5-*n*-butyl-2-norbornenes).

Olefin/Catalyst Ratio		Characteristics of Metathesis Products				Characteristics of Hydrogenated Products		
[ButNB]/[CTA] + [Gr2]	[ButNB] + [CTA]/ [Gr2]	№	*M*_n_ ^c^, kDa	*Ð* ^c^	*P*	№	*M*_n_ ^c^, kDa	*Ð* ^c^
6.9 ^a^	2.6 × 10^3^	**M7**	2.14	1.6	14	**H7**	2.02	1.7
5.0 ^a^	3.4 × 10^3^	**M5**	1.29	1.8	8	**H5**	1.44	1.7
4.0	4.1 × 10^3^	**M4**	1.15	2.0	7	**H4**	1.17	2.0
2.9	4.5 × 10^3^	**M3**	1.07	2.0	7	**H3**	1.11	2.0
2.5	4.6 × 10^3^	**M2.5**	0.80	1.9	5	**H2.5**	0.82	1.9
2.0 ^a,b^	4.6 × 10^3^	**M2**	0.60	1.7	3	**H2**	0.56	1.6

The CTA was hexene-1. The reaction mixture was cooled with ice in a water bath for the first 30 min of the reaction. ^a^ At room temperature. ^b^ Data obtained previously [30]. ^c^ The molecular weight (*M*_n_) and dispersity (*Ð*) values were determined from GPC data.

**Table 4:** Kinematic viscosities and tribological performance of PAO oils.

Sample	*v*_40 °C_, mm^2^/s	*v*_100 °C_, mm^2^/s	*f*	*D*_scar_, μm
**PAO-4**	14.3	3.61	0.104 ± 0.003	407 ± 14
**PAO-20**	137.0	19.1	0.103 ± 0.003	352 ± 32
**PAO-80**	674.7	77.7	0.086 ± 0.004	373 ± 12

**Table 5:** Elemental composition of the initial ball surface and the surface worn in the presence of poly(5-*n*-butyl-2-norbornene) oils.

№	Elemental Composition							
	C		O		Si		Fe	
	Weight Fraction, %	Atom Fraction, %	Weight Fraction, %	Atom Fraction, %	Weight Fraction, %	Atom Fraction, %	Weight Fraction, %	Atom Fraction, %
Initial surface ^a^	2.0 ± 0.5	8.7 ± 2.0	0.0	0.0	0.31 ± 0.04	0.58 ± 0.06	97.7 ± 0.5	90.7 ± 2.0
**M7**	2.1	8.4	4.4	12.8	0.27	0.45	93.2	78.4
**H7**	3.1	11.4	5.1	14.4	0.29	0.47	91.5	73.7
**PAO-4**	4.5	18.0	0.1	0.3	0.29	0.50	95.1	81.1

To facilitate comparison, the total sum of the weight or atom fractions of C, O, Si, and Fe atoms was normalized to 100% for all samples. ^a^ The standard deviation was determined for three replicate measurements.

**Table 2:** Density of hydrogenated and metathesis poly(5-*n*-butyl-2-norbornene) oils.

*T*, °C	M2.5	M3	M4	H2.5	H3	H4
10	0.899	0.909	0.911	0.887	0.898	-
20	0.888	0.898	0.901	0.877	0.888	0.890
40	0.866	0.877	0.879	0.855	0.867	0.869

**Table 3:** Rheo-tribological properties of poly(5-*n*-butyl-2-norbornene) oils.

Sample	*T*_1000 Pa·s_, °C	*η*_−40 °C_, Pa·s	*η*_40 °C_, mPa·s	*η*_100 °C_, mPa·s	VI	*E*_0 °C_, kJ/mol	*E*_75 °C_, kJ/mol	SAE J300 Grade	SAE J306 Grade	*f*	*D*_scar_, μm
**M2** ^a^	−66.3	7.49	25.7	4.44	129	40.9	27.0	0W-8	70W	0.111 ± 0.004	359 ± 22
**M2.5**	−53.7	66.6	68.9	9.39	141	48.4	30.8	15W-30	75W-85	0.093 ± 0.003	254 ± 21
**M3**	−37.2	1900	229	23.5	147	60.5	37.0	25W	80W-140	0.079 ± 0.001	260 ± 19
**M4**	−33.3	4800	345	35.9	168	62.5	36.1	too viscous	85W-250	0.087 ± 0.001	238 ± 20
**M5**	−22.8	97,000	761	59.0	158	75.1	42.2	too viscous	85W-250	0.088 ± 0.001	293 ± 33
**M7**	−7.3	10^8^	3380	142	145	102.1	44.6	too viscous	250	0.094 ± 0.002	307 ± 17
**H2** ^a^	−57.3	38.4	44.9	5.92	103	49.5	29.6	10W-12	75W	0.089 ± 0.001	203 ± 50
**H2.5**	−42.1	640	143	16.8	150	58.7	34.8	25W-50	80W-110	0.075 ± 0.002	265 ± 65
**H3**	−24.0	52,000	610	34.1	101	77.6	43.2	too viscous	85W-250	0.083 ± 0.001	125 ± 8
**H4**	−21.9	90,000	890	46.3	109	76.7	45.1	too viscous	250	0.077 ± 0.001	213 ± 17
**H5**	−11.8	4 × 10^6^	1920	69.7	100	96.3	50.1	too viscous	250	0.082 ± 0.001	118 ± 4
**H7**	3.9	>10^9^	11,900	334	157	118.8	56.4	too viscous	250	0.088 ± 0.001	289 ± 15

^a^ Data for **M2** and **H2** were obtained previously [30].

## Data Availability

The original contributions presented in the study are included in the article; further inquiries can be directed to the corresponding authors.

## Supplementary Materials

The following supporting information can be downloaded at https://www.mdpi.com/article/10.3390/polym17243333/s1. Figure S1: SEM and EDS images of unworn surfaces; Figure S2: SEM and EDS images of the wear spot surfaces of **M7** after point-on-point contact tests on a four ball tester; Figure S3: SEM and EDS images of the wear spot surfaces of **H7** after point-on-point contact tests on a four ball tester; Figure S4: SEM and EDS images of the wear spot surfaces of **PAO-4** after point-on-point contact tests on a four ball tester.
