# Supplementary material for: Low-Temperature Performance and Tribological Properties of Poly(5-n-butyl-2-norbornene) Lubricating Oils: Effect of Molecular Weight and Hydrogenation on the Viscosity and Anti-Wear Activity
Source: Polymers (Basel). 2025 Dec 17;17(24):3333. doi: 10.3390/polym17243333 (PMC12737209; doi:10.3390/polym17243333)
Supplement: Supplementary file 1 [file polymers-17-03333-s001.zip › polymers-4023275-supplementary.pdf]

Supplementary Materials

Low-Temperature Performance and Tribological Properties of Poly(5-*n*-butyl-2-norbornene) Lubricating Oils: Effect of Molecular Weight and Hydrogenation on the Viscosity and Anti-Wear Activity

Valeriia R. Nazemutdinova [1], Sergey O. Ilyin [1], Aleksandr A. Morontsev [1,*], Igor S. Makarov [1], Alyona I. Wozniak [1,2] and Maxim V. Bermeshev [1,2,*]

[1] A.V. Topchiev Institute of Petrochemical Synthesis, Russian Academy of Sciences, 29 Leninskiy pr., 119991 Moscow, Russia
[2] Enikolopov Institute of Synthetic Polymeric Materials, Russian Academy of Sciences, Profsoyuznaya str. 70, 117393 Moscow, Russia
* Correspondence: morontsev@ips.ac.ru (A.A.M.); bmv@ips.ac.ru (M.V.B.)

SEM and EDS images

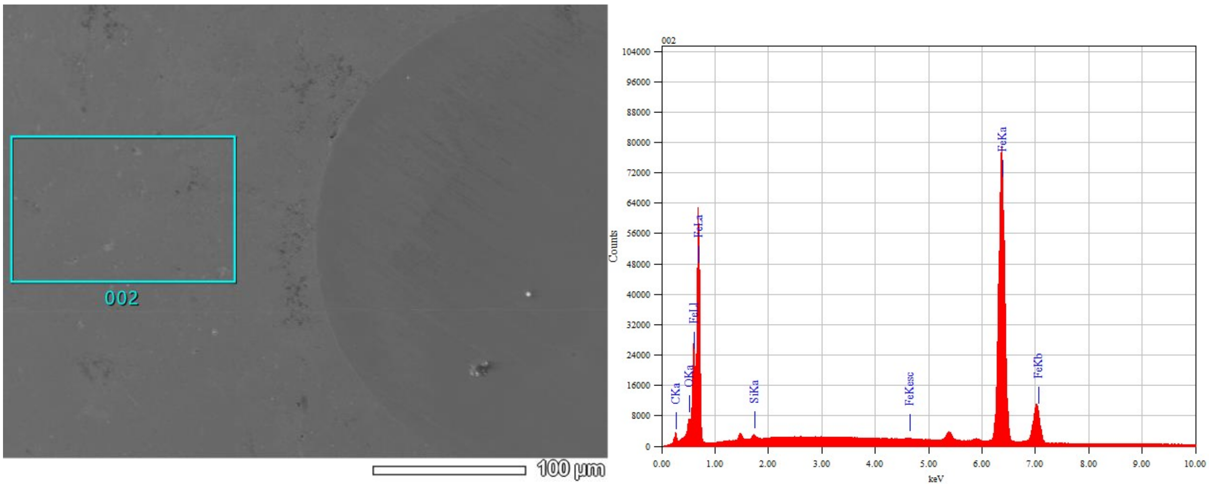

Figure S1. SEM and EDS images of unworn surfaces.

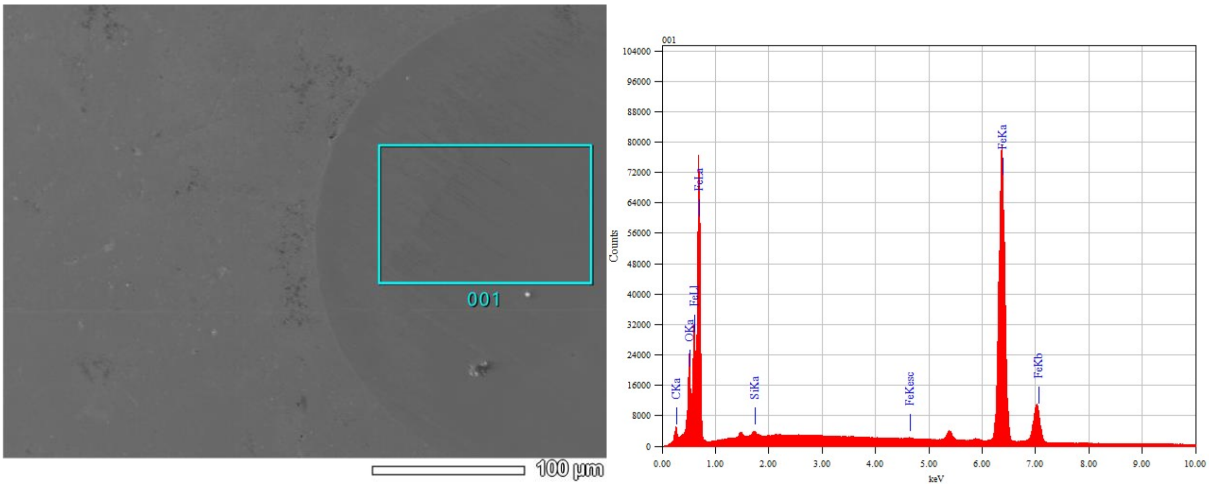

Figure S2. SEM and EDS images of the wear spot surfaces of M7 after point-on-point contact tests on a four ball tester.

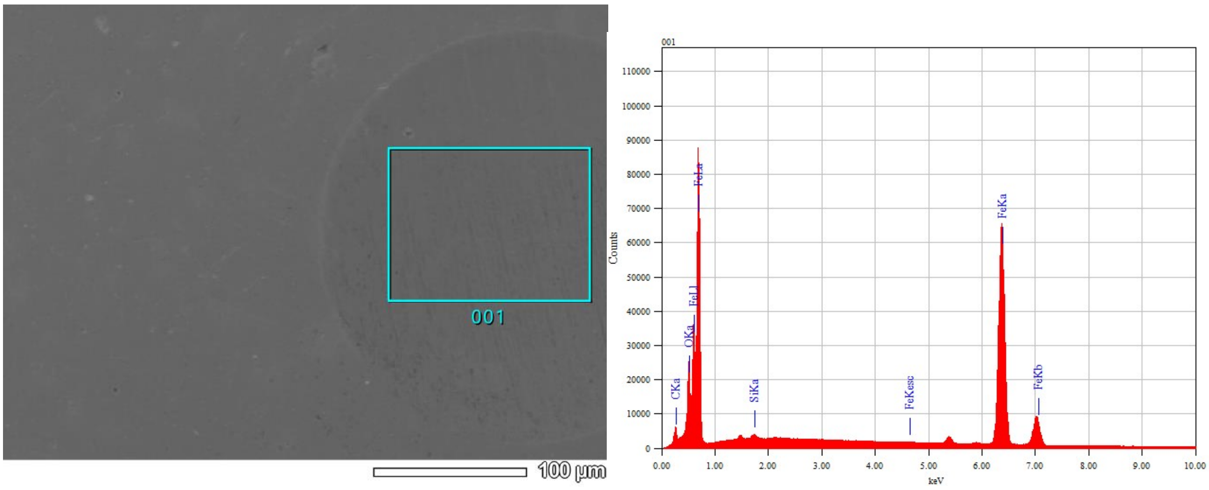

Figure S3. SEM and EDS images of the wear spot surfaces of H7 after point-on-point contact tests on a four ball tester.

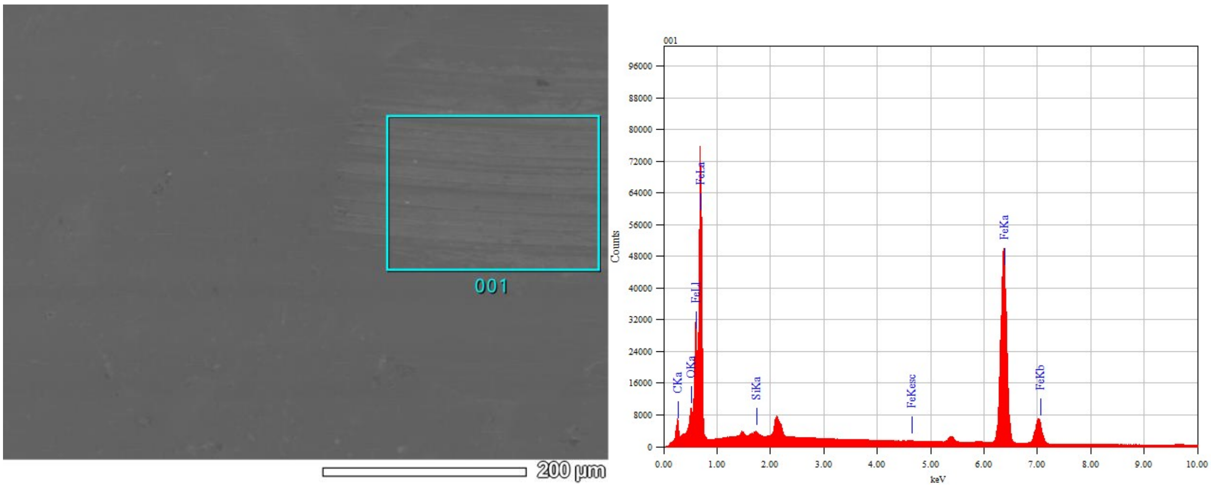

Figure S4. SEM and EDS images of the wear spot surfaces of PAO-4 after point-on-point contact tests on a four ball tester.
